# Spinal Cord Injuries Secondary to Mountain Biking Accidents — A Cause for National Alarm

**DOI:** 10.1089/neur.2024.0103

**Published:** 2024-11-11

**Authors:** William Chu Kwan, Pedram Laghaei, Harsh Kahlon, Tamir Ailon, Raphaële Charest-Morin, Charlotte Dandurand, Scott Paquette, Nicolas Dea, John Street, Charles G. Fisher, Vanessa Noonan, Marcel F. Dvorak, Brian K. Kwon

**Affiliations:** ^1^ Vancouver Spine Surgery Institute, Vancouver, Canada.; ^2^ Vancouver Spine Research Program (VSRP), Vancouver, Canada.; ^3^ Department of Surgery, Division of Neurosurgery, University of British Columbia, Vancouver, Canada.; ^4^ Department of Orthopaedics, University of British Columbia, Vancouver, Canada.; ^5^ International Collaboration on Repair Discoveries (ICORD), University of British Columbia, Vancouver, Canada.; ^6^ Praxis Spinal Cord Institute, Vancouver, Canada.

**Keywords:** mountain biking, health economics, spinal cord injury

## Abstract

While much attention in North America has been placed on hockey and other high impact sports as causes of spinal cord injury (SCI), over the past two decades, our Level 1 trauma center has experienced a much higher number of SCI from off-road mountain biking (MTB). Here, we aimed to characterize the epidemiology of SCI secondary to MTB, and we also sought to estimate the direct and other economic costs to assess their societal impact. A retrospective review was conducted of patients with SCI from MTB who were treated at our Level 1 trauma center between 2008 and 2022. Injury details were compiled, and we calculated the associated lifetime direct and other costs. Over the 14-year period, we identified 58 individuals (average age 35.5 years, 93% male) who suffered SCI while MTB. Twenty-seven suffered motor complete SCI (14 tetraplegia, 13 paraplegia) with estimated average lifetime costs in Canadian Dollars of $4.8 M and $4.5 M each, respectively. Thirty-one suffered motor incomplete SCI (26 tetraplegia, 5 paraplegia) with estimated average lifetime costs of $2.4 M and $1.6 M each, respectively. The total estimated lifetime costs for this group of SCI individuals were $195.4 M. From 2008 to 2022, we identified an SCI from MTB accidents at a rate of 4 patients per year. Our data underscores the urgent need for increased awareness and preventive measures to reduce the incidence of these devastating injuries, particularly in regions where MTB is prevalent.

## Introduction

Mountain biking (MTB) has evolved into a globally recognized sport, facilitated by lift-equipped bike parks at mountain resorts providing access to more challenging terrains.^1^ As of 2015, over 162 ski resorts in North America offer MTB during the summer, becoming a focus for advancing tourism and local economies.^2,3^ Unlike road cycling, MTB refers to off-road cycling demanding balance, agility, and adaptability in uneven terrain with specially designed bicycles equipped to handle diverse landscapes. With the exhilaration of high speed comes the inherent risk of injuries, with an incidence reported at 0.6% per year and 16.8 injuries per 1000 h of biking.^4,5^ Most injuries, such as contusions and lacerations, are relatively minor; however, severe injuries resulting in surgical intervention or even death have been reported.^6,7^

SCI is a life-altering condition profoundly affecting the individual, their families, and the community.^8,9^ The effects of SCI depend on the severity of neurological impairment and the level at which the injury occurs. Thoracic level injuries affect the lower extremity causing “paraplegia,” while cervical level injuries affect both upper and lower extremities causing “tetraplegia.” “Complete” SCI (AIS A) is the absence of motor and sensory functions, while “incomplete” SCI denotes some preservation of sensory (AIS B) or motor (AIS C or D) function below the injury.^10^ For example, patients with “incomplete tetraplegia” have absence of motor and sensory functions in both upper and lower extremities. The affected individuals experience a spectrum of physical, mental, and psychosocial sequelae including paralysis, debilitating chronic pain, mood disorders, and suicide.^11,12^ Caregiver’s physical, mental, and social burdens persist over decades.^8^

In addition, SCI incurs significant economic impact.^13,14^ Accurately capturing all direct and other costs of medical care, lost productivity, and caregiver requirements over a lifetime can be challenging. We previously published a 13-year (1995–2007) retrospective study characterizing the epidemiology of SCI secondary to MTB in British Columbia (BC), Canada.^15^ Here, we aim to provide an updated descriptive analysis of the epidemiology and highlight the economic impact through a detailed calculation of lifetime direct and other costs.

## Methods

### Study design and population

This retrospective cohort study was designed in accordance to STROBE guidelines.^16^ Institutional ethics approval was obtained prior to study initiation. Patients with acute SCI were identified within the Rick Hansen Spinal Cord Injury Registry (RHSCIR), a Canadian-wide observational database of patients with SCI.^17^ The study included patients with a diagnosis of SCI and an injury mechanism related to off-road MTB from March 2008 to July 2022, admitted our Level 1 Trauma Centre and regional referral center for all SCI in BC, serving a population of 4.3 M (2008) to 5.3 M (2022).^18^ Excluded were individuals who suffered a spinal column injury/fracture without SCI. We conducted a chart review to capture baseline demographics (age, sex, relationship status, occupation, comorbidities, Glasgow Coma Scale, associated injuries, American Spinal Injury Association Impairment Scale [AIS] at presentation and final follow-up, and spinal fracture diagnosis), accident characteristics (time of injury, mechanism [over the bars vs. collision with objects], and use of protective gears [helmet only, helmet and armour vs. none]), and aspects of intervention and hospital admission (treatment method [surgery vs. conservative], length of stay [LOS], adverse events [AE], and associated injuries).

### Cost analysis

The cost analysis was calculated for each patient based on hospital records. All costs are expressed in Canadian Dollars. For each patient, the total cost of SCI was divided into three parts: 1) initial direct costs including injury treatment, hospitalization, and rehabilitation costs, 2) lifetime direct costs including all costs directly related to the SCI after the initial treatment, and 3) other economic costs including loss of productivity to SCI and loss of quality-adjusted life year (QALY).

### Initial direct costs

For surgical treatment calculations, patients were categorized into three groups: cervical spine surgery, thoracic and/or lumbar surgery, and SCI without surgical intervention.^19^ For each patient, data for LOS in the acute-care hospital (both in the intensive care unit [ICU] and ward) and in the rehabilitation center were obtained, along with any AEs. For all above-mentioned costs, the per diem Canadian Institute for Health Information (CIHI) cost estimator was used to obtain the average costs in 2019 dollar-cost figures.^19^ These costs were updated to 2023 dollars using the inflation calculator from the Bank of Canada (BoC).^20^ The initial direct cost was calculated by multiplying LOS for each patient by the daily average cost for ICU, general ward, and acute rehabilitation and adding the cost of treating AEs.^21^

### Lifetime direct costs

To calculate lifetime direct costs, patients were classified as complete or incomplete paraplegia or tetraplegia. We estimated the life expectancy of each patient using the ratio of life expectancy for persons with SCI versus the general population.^22^ Krueger and associates calculated an average cost per year for each type of injury: acute care rehospitalizations, day procedures, health care providers, pharmaceuticals, institutional care, caregiving services, and home adaptations/adaptive equipment/vehicle modifications.^23^ The Krueger costs are in 2019 dollars and were brought to 2023 dollars using the BoC inflation calculator,^20^ except for caregiving services, which we adjusted by using the current hourly cost of $42 and the average number of weekly caregiving services (paraplegia = 5 h, tetraplegia = 70 h) as provided by Praxis Spinal Cord Institute through a consultative process of persons living with spinal cord experience (PLEX).^20^ For each patient, we calculated a direct cost for each year of life which were then discounted at a real discount rate (net of inflation) of 1.5% and summed to obtain a present value total direct cost.^24^

### Other costs

To estimate the value of lost productivity, we multiplied the average yearly income for workers in Canada by the estimated percentage income reduction for each type of injury, for each year until the patient reached age 66.^25^ The percentage of income reduction (Table 1) was provided by PLEX through deliberations within the PLEX team at Praxis Spinal Cord Institute. Loss of QALYs estimation was done in two parts as described by H. Krueger.^23^ First, the years of life lost that were calculated as the difference between the life expectancy with SCI and the general population life expectancy at the age of injury. Next, the loss of quality of life was estimated for each year with SCI using the disability weights calculated by H. Krueger,^23^ (Table 1) reported by productivity loss estimates and QALYs lost were discounted at 1.5% yearly to adjust for the diminished value over time relative to present value.

**Table 1.** Percentage of Productivity Loss and Disability Weight by Type of Spinal Cord Injury as Described by H. Krueger^22^

Type of injury	Incomplete paraplegia	Complete paraplegia	Incomplete tetraplegia	Complete tetraplegia
Productivity loss	33%	40%	45%	75%
Disability weight	0.059	0.296	0.228	0.598

## Results

### Baseline demographics

**FIG. 1.**
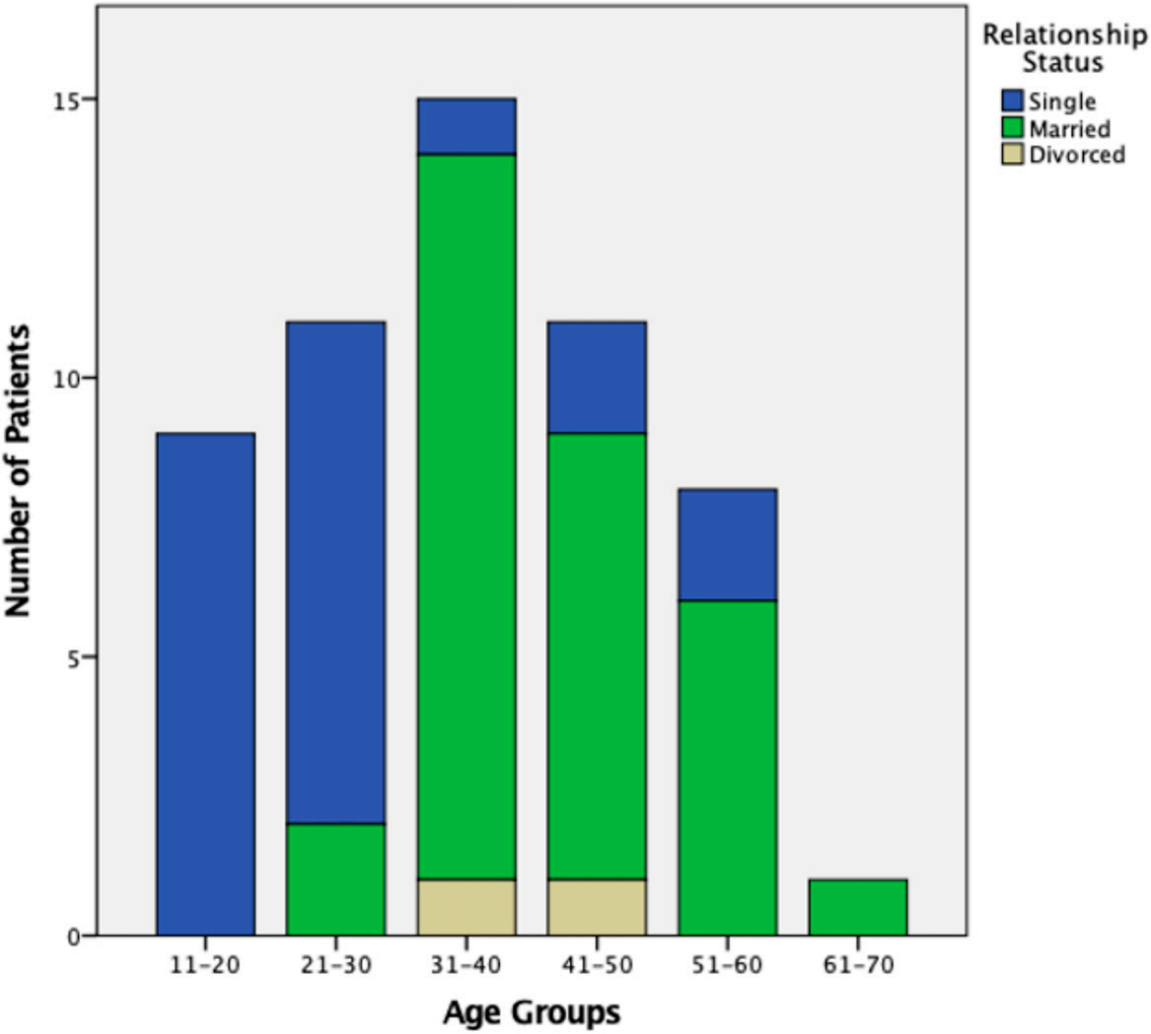
Distribution of age and relationship status of patients with spinal cord injury. Twenty-three (41.8%) patients were single; 30 (54.5%) were married or in common law relationships; 2 (3.6%) were separated or divorced.

We identified 54 male (93.1%) and 4 female (6.9%) patients with SCI. The mean age was 35.5 (SD = 13.1). Relationship status and age distribution were available for 55 patients (Fig. 1). Employment status was available for 54 patients (Table 2). This was a fairly healthy group of individuals with few medical comorbidities such as asthma (*N* = 4, 7%) and previous fracture (*N* = 2, 3%). There was only one instance (*N* = 1, 2%) of each of the following comorbidities: oncology, depression, osteoarthritis, osteoporosis, atrial fibrillation, esophageal reflux, dyslipidemia, neuropathic pain, gastroenteritis, and scoliosis. There was no patient with myocardial infarction, congestive heart disease, cerebrovascular disease, liver disease, diabetes mellitus, dementia, nor connective tissue disease.

**Table 2.** Distribution of Employment

Employment or profession	*N*	%
Professional specialty	14	24.1
Precision, production, craft and repair	11	19.0
Student	6	10.3
Executive, administrative, and managerial	6	10.3
Technicians and related support	4	6.9
Others^a^	13	22.3
Status unavailable	4	6.9

^a^ Others: Sales (*N* = 3), Machine operators (*N* = 3), Not working (*N* = 2), Profession bikers (*N* = 2), Administrative support (*N* = 1), Farming (*N* = 1), Laborer (*N* = 1).

### MTB details

The geographical location of all patients was available, with 21 individuals (36%) biking in Whistler, BC, one of the largest and most popular resorts in North America located 2 hours from Vancouver, accessible by public transportation, and with chair-lift capabilities to carry up one’s bike up the gondola lift for bikers to ride down. The mechanism of injury was available for 57 patients (1 unavailable). Forty-five (77.5%) were propelled over the handlebars. Seven (12.1%) sustained their injury in a collision, while 5 (8.8%) suffered miscellaneous mechanisms. Data on usage of protective gear was available for 44 patients (14 unavailable), with 38 (86.3%) wearing only helmets, 4 (9.1%) wearing helmets and body protection, and 2 (4.5%) not wearing any protection.

### SCI characteristics

The baseline severity of neurological impairment was classified on the American Spinal Injury Association Impairment Scale (AIS).^10^ 21 (36.2%) were AIS A, 6 (10.3%) were AIS B, 11 (19.0%) AIS C, and 20 (34.5%) AIS D reflecting the fact that almost half (45%) suffered a “motor complete” paralysis (AIS A or B) (Table 3). The majority of SCIs were sustained in the C3–C7 cervical spine (*N* = 39, 67.2%), followed by thoracolumbar injuries (*N* = 16, 26.6%), and C1–C2 injuries (*N* = 3, 5.2%). Table 4 provides further detail on the AIS level distribution and conversion from admission to discharge. The most common associated injuries were thoracic/pulmonary injuries (*N* = 21, 36%), upper extremity fractures (*N* = 21, 36%), and facial lacerations/fractures (*N* = 20, 34%). There were no reported deaths during admission and follow-up period.

**Table 3.** American Spinal Injury Association Impairment Scale of SCI at Presentation

AIS grade	Type of injury	Description	AIS at presentation
A	Complete	No sensory or motor function is preserved in the sacral segments S4-S5.	21 (36.2%)
B	Sensory Incomplete	Sensory but not motor function is preserved below the neurological level and includes the sacral segments S4-S5 (light touch, pin prick at S4-S5 or deep anal pressure), AND no motor function is preserved more than three levels below the motor level on either side of the body.	6 (10.3%)
C	Motor Incomplete	Motor function is preserved below the neurological level and more than half of key muscle functions below the single neurological level of injury (NLI) have a muscle grade less than 3.	11 (19.0%)
D	Motor Incomplete	Motor function is preserved below the neurological level and at least half of key muscle functions below the NLI have a muscle grade of 3 or greater.	20 (34.5%)

AIS, American Spinal Injury Association Impairment Scale; SCI, spinal cord injury.

**Table 4.** Conversion of AIS Level from Admission to Discharge

	Discharge				
Baseline	A	B	C	D	E
A	15	4	2	0	0
B	2	2	2	1	0
C	0	1	0	9	0
D	0	0	0	20	0

### Cost analysis

**FIG. 2.**
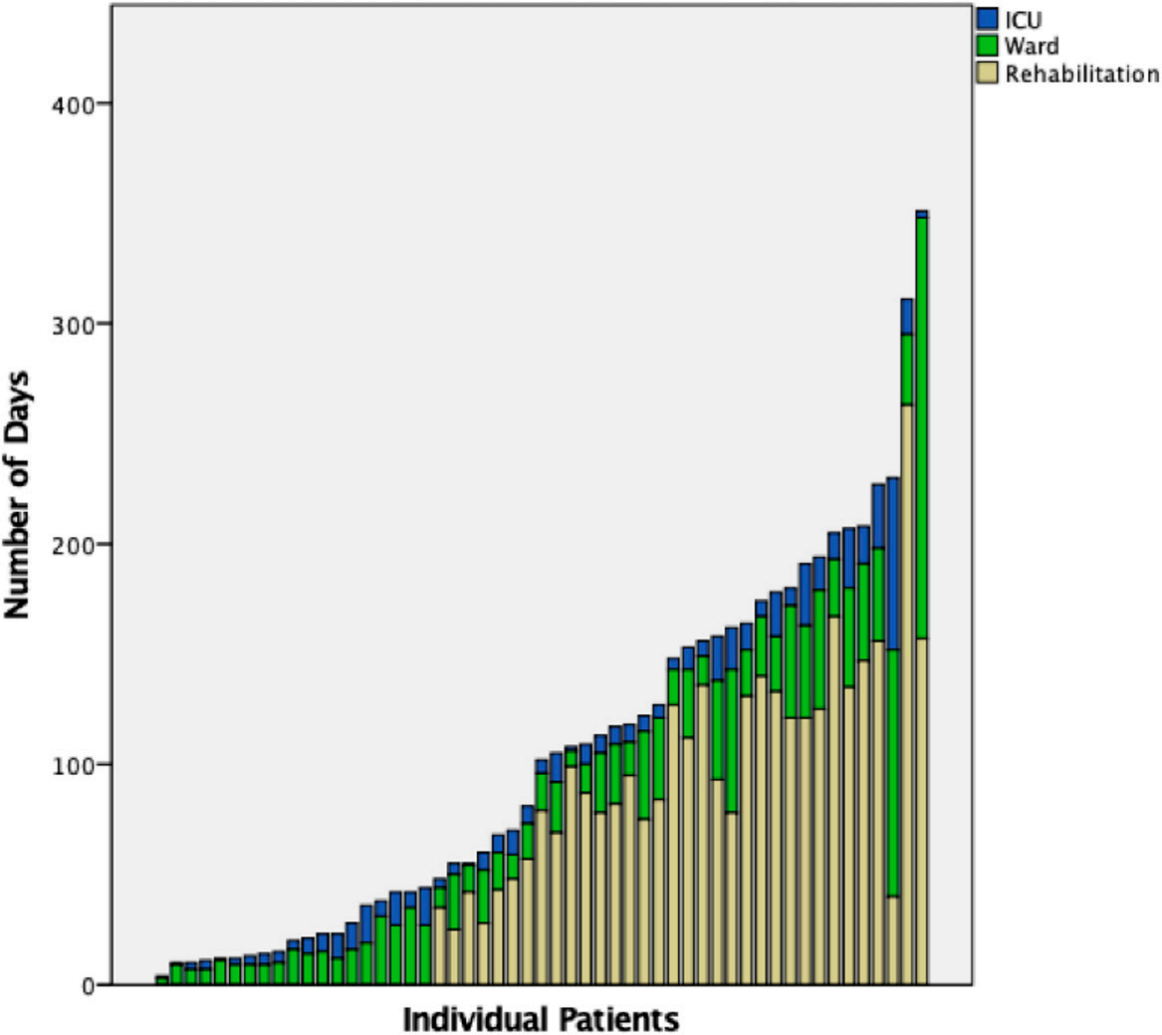
Individual patient graphical representation for length of stay (LOS) for ICU, ward, and rehabilitation.

Of the 42 cervical SCI, 34 (81%) had surgery. Of the 16 thoracolumbar injuries, 14 (88%) had surgery; a total of 10 (17.2%) patients did undergo surgical management as 9 were AIS D and 1 was AIS A. For ICU, ward, and rehabilitation, the average LOS was 10.9 (SD = 11.6), 26.5 (SD = 28.7), and 102.8 (SD = 48.41) days, respectively. The cumulative LOS were 577, 1539, and 3494 days, respectively. Figure 2 depicts LOS for each patient. Table 5 summarizes intraoperative and post-operative AE. Table 6 provides the calculations for lifetime direct and other costs models for an individual patient with either incomplete paraplegia (*N* = 5), complete paraplegia (*N* = 13), incomplete tetraplegia (*N* = 26), or complete tetraplegia (*N* = 14). The average combined cost (acute direct, lifetime direct, and lifetime other) was $1.6 M, $2.4 M, $4.5 M, and $4.8 M, for incomplete paraplegia, incomplete tetraplegia, complete paraplegia, and complete tetraplegia, respectively. The total combined cost for these groups were $8.1 M, $61.3 M, $58.6 M, and $67.3 M, respectively.

**Table 5.** List of Adverse Events and Cost Utilization Calculated Using the CIHI Cost Estimator

	Group 1 (SCI)	
	*N*	Total cost
Dural Tear	2	3,746
Pneumonia	15	14,040
Pulmonary Embolism	3	2,580
Ventilator	3	6,885
Atelectasis	7	4,543
Pleural Effusion	10	9,300
Transfusion	3	1,845
MI	7	9,632
DVT	2	1,788
Wound Complication	5	17,125
UTI	22	32,230
Bowel Obstruction	3	1,929
Neuropathic pain and autonomic dysreflexia	36	34,452
Nephrolithiasis	4	3,188
Pain Crisis	8	5,288
Orthostatic Hypotension	10	4,700
Delirium	5	6,080
Pressure Ulcer	5	3,995
Ventilator	4	9,180
Others	25	27,796
Total Cost		200,322

CIHI, Canadian Institute for Health Information; DVT, Deep vein thrombosis, MI, Myocardial infarction; SCI, spinal cord injury; UTI, Urinary tract infection.

**Table 6.** Summary of Costs for Each Population Groups as Calculated in This Study

	Incomplete paraplegia	Incomplete tetraplegia	Complete paraplegia	Complete tetraplegia	Total
Number of Patients	5	26	13	14	58
Initial Direct Cost (Average)	$ 53.3 K	$ 93.6 K	$ 112.5 K	$ 245.0 K	
Initial Direct Cost (Total)	$ 266.6 K	$ 2.4 M	$ 1.5 M	$ 3.4 M	$ 7.6 M
Lifetime Direct Cost (Average)	$ 1.18 M	$ 1.8 M	$ 3.9 M	$ 4.0 M	
Lifetime Direct Cost (Total)	$ 5.9 M	$ 47.2 M	$ 50.3 M	$ 55.6 M	$ 159.1 M
Lifetime Indirect Cost (Average)	$ 387.8 K	$ 448.0 K	$ 524.0 K	$ 589.2 K	
Lifetime Indirect Cost (Total)	$ 1.9 M	$ 11.6 M	$ 6.8 M	$ 8.2 M	$ 28.6 M
Total Cost Per Patient	$ 1.6 M	$ 2.4 M	$ 4.5 M	$ 4.8 M	
Total Cost Per Population	$ 8.1 M	$ 61.3 M	$ 58.6 M	$ 67.3 M	$ 195.3 M

## Discussion

In this retrospective study, we described a high incidence of individuals living with SCI from MTB in BC over a 14-year period (2008–2022) and provided a comprehensive analysis of the associated economic burden. With a total of 58 patients, most were male with an average age of 35.5. More than half of the patients (*N* = 30, 52%) were married, causing economic stressors to partners and families. The majority of single patients (*N* = 18, 78%) were under 30 years old, causing stressors to caregivers. Two-thirds of patients (38 of 58, 66%) suffered AIS A, B, or C injuries, representing a degree of neurological impairment that would certainly affect the ability to walk, bladder and bowel control, and sexual function. Surgical treatment was necessary for 48 (82.8%) patients. The cumulative LOS in ICU, ward, and rehabilitation for all patients were 577, 1539, and 3494 days, respectively. The total combined cost for incomplete paraplegia, complete paraplegia, incomplete tetraplegia, or complete tetraplegia groups were $8.1 M, $61.3 M, $58.6 M, and $67.3 M, respectively. Our analysis showed that while the initial direct costs associated with paraplegia are lower compared with quadriplegia, the lifetime costs for individuals with paraplegia are comparable to those with quadriplegia. This is primarily due to a greater loss of productive years among the paraplegic group. Consequently, the total cost for paraplegics is only marginally less than that for quadriplegics. The total cost of 58 patients amounted to $195.4 M. We found that the highest economic burden was due to the lifetime direct cost attributed to the younger age of patients.^26^

When reading these observations about SCI in MTB, it is only natural to consider how this compares to the incidence of SCI in other recreational/athletic activities, particularly those that attract much public attention. It is particularly striking to us as a Level 1 trauma center within Canada — a country where ice hockey draws considerable public interest — that during this 14-year study period, we treated only 3 individuals who suffered SCIs from playing ice hockey (average of 0.21 SCI/year) in comparison to the 58 patients who were paralyzed while MTB (average of 4.1 SCI/year). Across the border in the United States where American football is passionately followed, the National Football League (NFL) reported 14 SCIs over 11 seasons between 2000 and 2010 (average of 1.3 SCI/year).^27^ The National Center for Catastrophic Sports Injury Research (NCCSIR) reported 76 “quadriplegic” and 17 “incomplete” SCI in high school and college football players over 13 years (average of 7.1 SCI/year).^28^ While at quick glance, this may seem like comparable (if not higher) incidences to our reported incidence of 4.1 SCI/year, it should be remembered that this represents the reporting of football injuries across the *entire country* of the United States, with a population base of 333 million.^29^ In contrast, our data reflects the injuries occurring each year within a *single Canadian province* (BC) of only 5.3 million people.^18^ If an entire country of 333 million witnesses 7.1 SCI from football each year (a sport known for its violent collisions), then a single province of 5.3 million treating 4.1 SCI yearly from MTB represents, in our opinion, a concerningly high incidence.

Dodwell et al. published a retrospective chart review of SCI from MTB and reported similar patient demographics (young, male, professional). Dodwell reported 43 SCI (AIS A = 18, AIS B = 5, AIS C = 10, AIS D = 10) over a 13-year span (average of 3.3 SCI/year). Meanwhile our study reported on 58 SCI (AIS A = 21, AIS B = 6, AIS C = 11, AIS D =20) over a 14.3-year span (average of 4.1 SCI/year).^15^ Whether this 3.3 to 4.1 SCI/year increase represents a truly statistically significant change, the reality is that MTB is growing in popularity, highlighting the need for preventative measures. Dodwell et al. reported that 14.4% were not wearing protective gear, while we found 4.5% were not wearing protective gear.^15^ The implementation of helmets with facial protection, impact-resistant lenses, and protective devices for trunk and extremities has demonstrated efficacy in reducing and preventing injuries, but not necessarily SCI.^7^ The most common associated injuries in our cohort were thoracic injuries, upper extremity fractures, and facial lacerations or fractures. This finding aligns with several reviews, which report the prevalence of thoracic injuries ranging from 4% to 49%, upper extremity injuries from 8% to 86%, and facial or head injuries from 6% to 54%.^6,7^

Our center is a tertiary-care referral center in a mountainous province where MTB is very popular. During a 6-month interval in Whistler, BC in 2009, there were 898 mountain bikers who suffered 1759 injuries.^1^ It is important to note the difference between an institutional-based incidence compared to a nationwide or population-based incidence. We acknowledge that our mountainous geography in BC, Canada, may be somewhat unique, so there will be other centers where such recreational activity and injury are uncommon. There are undoubtedly other ski-resorts in Canada and the US where mountain biking is popular in the summer months, and we would surmise that something of a similar nature is occurring there. We are not aware of any other publication that specifically addresses the incidence of SCI after mountain biking and the broad economic impact that this injury has.

The risk of SCI due to MTB is worrisome given the growing popularity of the sport.^30^ While we did close the inclusion period for this current study in 2022, in review of the same local prospective SCI registry that was used for this paper, we have documented another 21 SCI due to MTB between 2022 and 2024. This is not to introduce “new data” to this paper, but rather to simply point out that what we experienced in MTB-related SCI between 2008 and 2022 was not an aberration, and we are continuing to see these catastrophic injuries at a similar (if not higher) rate. While to our knowledge there has not been a recent publication that has focused specifically on SCI in mountain-bikers, the increasing popularity of the sport and access to mountain-biking parks across North America would make it unlikely that what we are observing is a phenomenon isolated to our province. The incidence of injuries in general has prompted local educational campaigns such as the Vancouver Coastal Health (VCH) “Shred Safe” campaign that ran in 2016 to reduce preventable MTB injuries.^31^ Currently, the BC government is actively invested in cycling safety and prevention efforts; however, these initiatives do not extend to MTB prevention.^32^ Advocacy efforts should emphasize the importance of appropriate rider education (technique training, familiarization of circuits, insight to operate within their capabilities, and fatigue awareness), safe and appropriate equipment (well-maintained high-quality bikes, no exposed handlebar ends, helmets, facial protection, gloves, and shin guards), and thoughtful racecourse design (speed limit, course evaluation).^5–7,33,34^

As mentioned earlier, there is a great public consciousness about contact sports that result in SCI — particularly ice hockey in Canada. Due to catastrophic cervical SCI resulting from cross-checking into the boards from behind, much awareness has been raised about the potential for these devastating injuries which has resulted in penalties for cross-checking from behind and the ubiquitous “STOP” signs being applied on the backs of practice jerseys. A drop in the incidence of hockey-related spinal injuries has been attributed to programs, such as the “Heads Up, Don’t Duck” campaign.^35^ Tator et al. reported from the Canadian Ice Hockey Spinal Injuries Registry a yearly average of 7.3 spine injuries across the nation between 2006 and 2011, with many of these suffering associated neurological injury.^36^ However, as noted earlier, during the 14**-**year span of this study, in our province alone, we have treated only 3 hockey players for SCI, in comparison to the 58 mountain bikers with SCI. If every year, 3-4 ice hockey players in our province (let alone our entire country) suffered severe cervical SCI while playing our “national pastime,” there would most certainly be alarms ringing from a public health standpoint. We contend that such awareness is required for MTB which — while far less popular than hockey — is resulting in immensely greater injury burden, not just for patients but for the health care system at large. Certainly, the impact that these injuries have had on these individuals and their families, alongside the significant health care utilization required for managing the physiological sequelae of SCI, make this an important public health care issue.^6,7,33^

It is important to acknowledge the limitations of our study design, particularly around the complexity of capturing the full economic impact of a condition such as SCI that incurs significant medical costs along a continuum of time, in addition to costs associated with lost employment and caregiver needs. Our cost estimations are based on the age of each of the 58 patients in the study at the time of injury, and an extrapolation of costs over each individuals’ life span. Given the differing degrees of impairment and resultant requirements, this study generalizes certain costs, especially the costs following discharge from acute care. In addition, despite being the only Level 1 spine trauma center in the province, it is possible that mountain bikers with less severe injuries were not transferred to our center. This would result in an underestimation of the true rate of SCI over this time period.

## Conclusion

Over a 14-year period (2008–2022) in our Level 1 Trauma Center in BC, Canada, we had 58 patients with SCIs due to MTB, leading to an estimated economic burden of $195.4 M (apart from the almost unquantifiable impact that the SCI had on the individuals themselves and their families). Considering the increasing popularity of MTB across Canada and the prevalence of bike parks in mountain resorts throughout the country, there is an urgent need to raise public awareness of SCI in MTB. This study also serves as a starting point for a much-needed dialogue with MTB parks and mountain bike associations to raise awareness and initiate partnerships that acknowledge the common desire to reduce the incidence of these catastrophic injuries.

## Abbreviations Used

AE: Adverse events
AIS: American Spinal Injury Association Impairment Scale
BC: British Columbia
BoC: Bank of Canada
CIHI: Canadian Institute for Health Information
DVT: Deep vein thrombosis
GCS: Glasgow Coma Scale
ICU: Intensive care unit
LOS: Length of stay
MI: Myocardial infarction
MTB: Mountain biking
NCCSIR: National Center for Catastrophic Sports Injury Research
PLEX: Persons living with spinal cord experience
QALY: Quality-adjusted life year
RHSCIR: Rick Hansen Spinal Cord Injury Registry
SCI: Spinal cord injury
SD: Standard deviation
UBC: University of British Columbia
UTI: Urinary tract infection
VCH: Vancouver Coastal Health
VGH: Vancouver General Hospital
